# Current Understanding and Future Directions of Transcatheter Devices to Assist Failing Fontan

**DOI:** 10.1016/j.jscai.2024.101334

**Published:** 2024-03-12

**Authors:** Nicola Pradegan, Tea Lena, Chiara Tessari, Michele Gallo, Vincenzo Tarzia, Alvise Guariento, Massimo Padalino, Vladimiro Vida, Gino Gerosa

**Affiliations:** a Cardiac Surgery Unit, Heart Transplant and MCS Program, Cardio-thoracic-vascular and Public Health Department, Padova University Hospital, Padova, Italy; b Department of Cardiothoracic Surgery, University of Louisville, Louisville, Kentucky; c Cardiac Surgery Unit, Cardio-Thoracic-Vascular and Public Health Department, University of Padova, Podova, Italy; d Pediatric Cardiac Surgery and Congenital Heart Disease Unit, Cardio-Thoracic-Vascular and Public Health Department, Padova University Hospital, Padova, Italy

**Keywords:** Fontan, mechanical circulatory support, transcatheter intravascular pumps

## Abstract

Even if the Fontan operation is the surgical treatment of choice in patients with univentricular physiology, it remains a palliative strategy. Consequently, when Fontan patients reach adulthood, the majority of them develop late clinical sequelae of a failing cavo-pulmonary circuit (eg, liver failure, protein-losing enteropathy, and arrhythmias). Although heart transplantation represents the gold standard to treat this condition, Fontan patients usually accede to this therapy late, when risk of mortality is significantly increased, and a shortage of donor hearts limits transplantation in this special population. Mechanical circulatory support is an emerging field, but it is still in the experimental stage. Current mechanical circulatory devices have been used in Fontan circulation but are associated with the need for high-risk redo surgery. Percutaneous pumps are an emerging field that is still under investigation, with multiple prototypes developed. This review aims to analyze the hemodynamic profile of the developed intravascular pumps and their application in the preclinical scenario in the Fontan circulation.

## Introduction

Since its first report in the early 1970s,^1^ the Fontan operation has been widely used for decades to palliate univentricular physiologies. To date, it is estimated that almost 70,000 patients have undergone Fontan circulation completion,^2^ and this population will double over the next 20 years.^3^ Even if this surgical strategy has led many children to reach adulthood, the Fontan operation alters the cardiopulmonary physiology. In fact, in a Fontan circulation, there is no pump to propel blood into the pulmonary arteries because the systemic veins are directly connected to the pulmonary arteries (with 2 vascular beds connected in series). The remaining postcapillary energy is harnessed to drive blood through the lungs; however, the pulmonary impedance hampers venous return through the pulmonary vascular bed, similar to any dam wall or bottleneck, leading to congestion upstream and restricted flow downstream.^4^ Consequently, the majority of the clinical and physiologic complications in a Fontan circuit are due to the upstream venous congestion and the downstream decreased output and generally result in a “failing Fontan,”^5^ which has a high incidence of mortality.^6^ Apart from specific cardiac Fontan-related complications (eg, subnormal resting cardiac output, impaired exercise capacity, increased risk of thromboembolism, and increased risk of arrhythmias), the long-term deleterious effects of substantial systemic venous hypertension include progressive dysfunction of other organ systems, particularly renal, lymphatic, gastrointestinal (GI), and hepatic systems.^7^

Even if heart transplantation (or combined heart–liver transplantation) might represent the gold standard to treat end-stage Fontan patients, it appears that many subjects with failed Fontan circulation may be denied heart transplantation because of a perceived impermissibly high risk of surgery due to its complexity and apparent multiorgan failure.^8^ Fontan patients undergo multiple reoperations, significantly increasing their overall mortality at the time of transplantation. Besides, the long-standing increased systemic venous pressure associated with the reduced pulmonary flow can produce microscopic liver and pulmonary changes that can become irreversible despite heart transplantation, with consequent higher mortality in this subgroup of patients.^9^ Indeed, the increasing donor shortage makes heart transplantation a very limited solution, for which alternative strategies are required.

Transcatheter pumps able to assist the Fontan circulation have been hypothesized for years, but despite their multiple advantages (surgical redo avoidance, subpulmonary ventricle effect restoration with potential reversibility on liver and lung anatomy and function), they have not been clinically tested yet. With this review, we aimed to analyze the current devices under preclinical investigation for assisting the failing Fontan circulation and restoring a right circulation.

## In vitro studies

**Figure 1:**
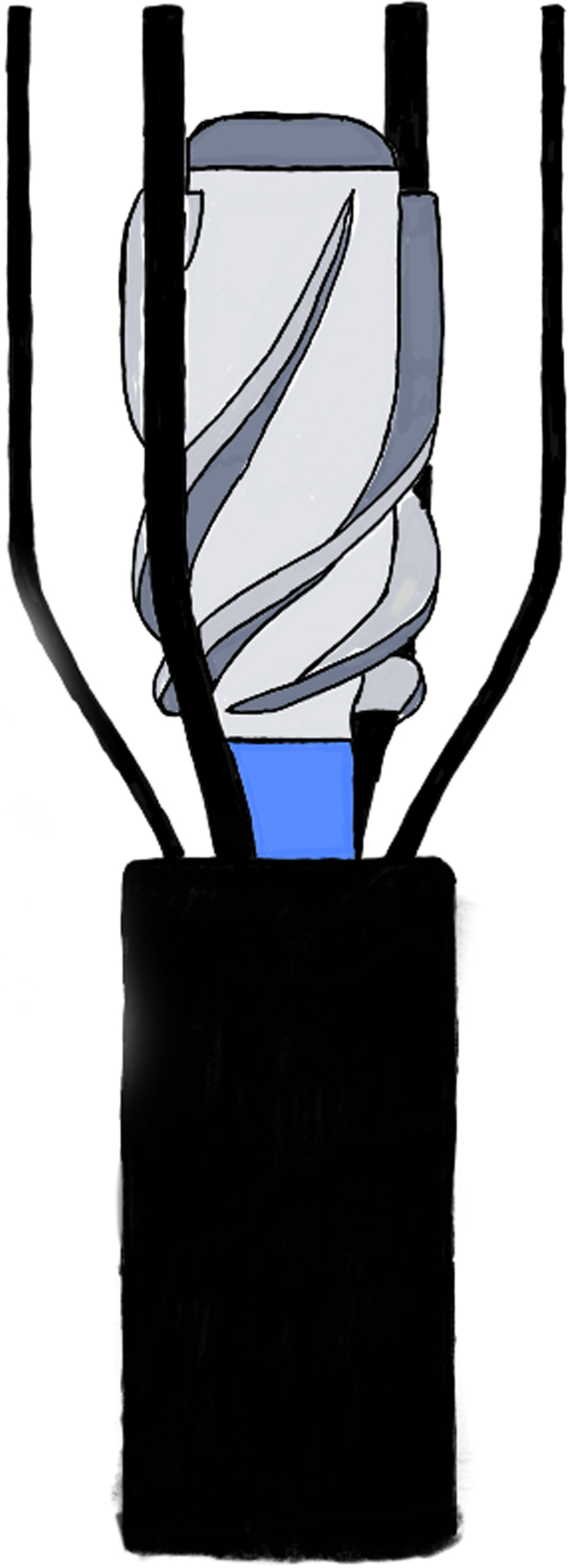
**Collapsible axial flow pump with radially arranged filaments**.

The first evaluated device was an intravascular axial flow blood pump.^10^ Throckmorton and Kishore^10^ designed a collapsible device for percutaneous positioning in the inferior vena cava (IVC) and/or superior vena cava (SVC). The outer protective cage was designed with radially arranged filaments, which were modified in the subsequent analysis with twisted filaments to get better performance^11^ (Figure 1). All models^10^^,^^12^^,^^13^ demonstrated an acceptable hydraulic performance by delivering 2 to 6 L/min at a rotational speed of 6000-10,000 rpm and generating a pressure rise of 5-20 mm Hg. The hemolysis profile of the design was also favorable. When testing this device both in an idealized Fontan^14^ and in real anatomical patient-based models,^15^^,^^16^ the above mechanical design was found to produce higher pressures, with a low effect of blood viscosity on the design function and a low probability of blood damage; indeed, the anatomical model with the pump-generated higher energy gains than the idealized model. Following these early results, 3-dimensional models were produced and tested.^17^ As a second step, the same research group tried to analyze the in vitro effect of 2 pumps (SVC + IVC) rather than 1 only in the IVC, and they found that 2 blood pumps positively augmented the hydraulic energy in the total cavopulmonary connection circuit as a function of flow rate and rotational speed, with also slightly more elevated damage indices (3.5% vs 2%) with this double configuration.^18^ Even if this model has been largely analyzed in vitro, applications have still to be performed. The last analysis was conducted using fluid-structure interaction studies,^19^ showing low-deformation parameters with materials, such as Nitinol, silicone, and steel.

**Figure 2:**
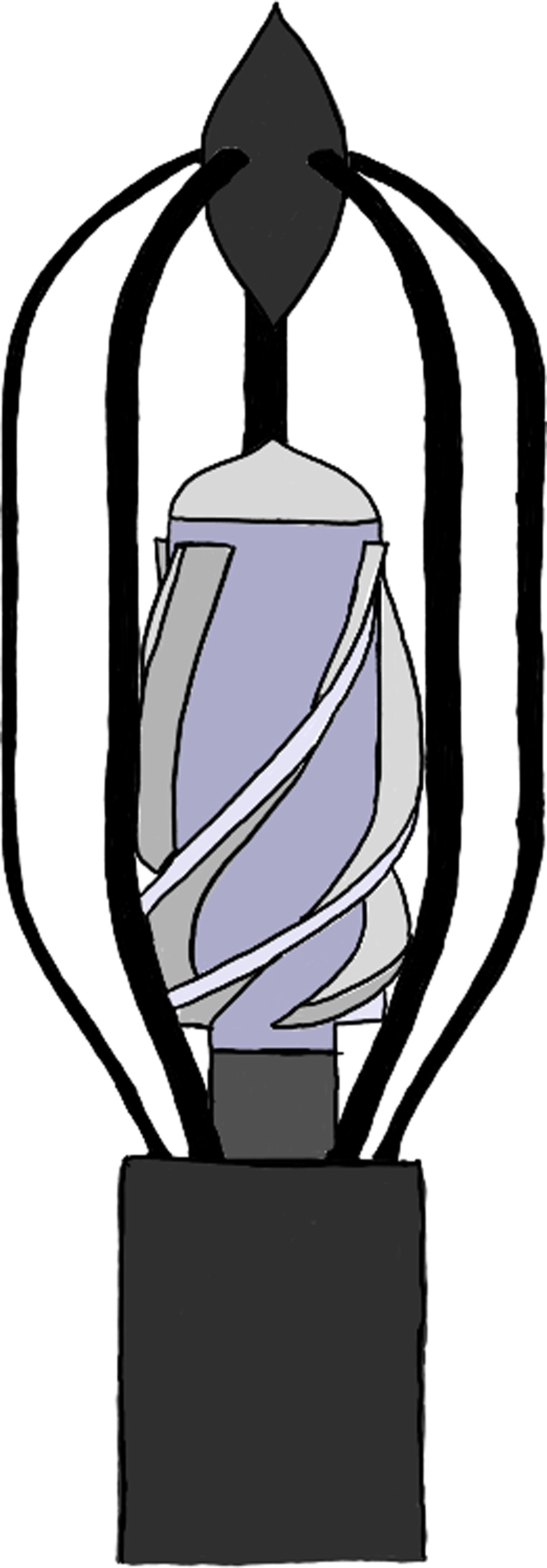
**Axial flow blood pump consisting of a protective cage of filaments, an impeller blade set, and an outlet nose cap**.

The second model^20^^,^^21^ was a new axial flow blood pump, with a new design consisting of a catheter, protective cage of filaments, impeller blade set, and outlet nose cap. The outer protective stent had radially arranged filaments that served as touchdown surfaces for the rotating impeller blades (Figure 2). Impeller rotation was generated through a drive cable-fluid seal combination with a port to supply a dextrose solution as a lubricant and to flush the fluid seal of accumulated blood elements. Among the different impeller geometries (blade twist angles between 100° and 600°), the impeller with 400° of blade twist outperformed the other designs, producing 3-31 mm Hg pressure increase for flow rates of 1 to 5 L/min for 6000 to 8000 rpm.^22^

**Figure 3:**
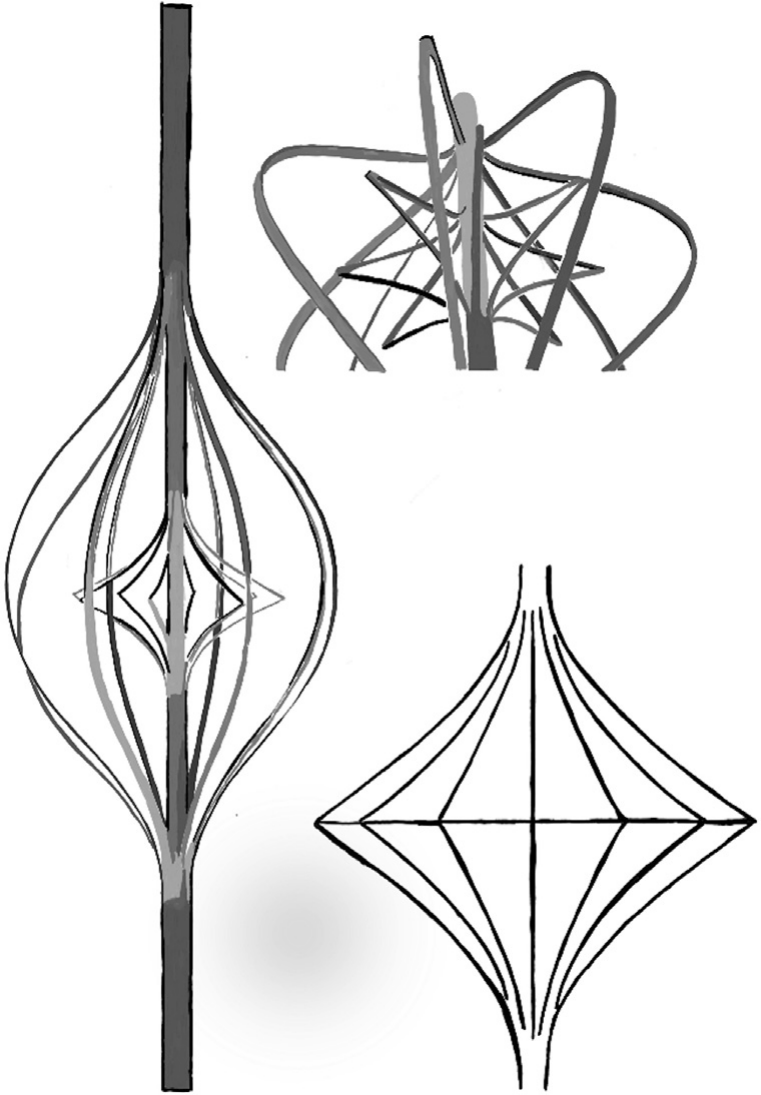
**Viscous impeller pump represented as a smooth 2-sided conical actuator disk with rotation in the vena caval axis**.

In 2010, Rodefeld et al,^23^ in parallel to the previous devices, hypothesized a viscous impeller pump, based on the von Karman viscous pump principle. In their first report, they conceptualized a pump in an idealized adult total cavo-pulmonary connection model of semiphysiologic flow. The impeller was represented as a smooth 2-sided conical actuator disk with rotation in the vena cava axis. A nonrotating actuator disc stabilized cavo-pulmonary flow, reducing power loss by 88% (Figure 3). Increase in pressure of 5 to 20 mm Hg and 0 to 5 L/min flow was obtained with a vaned impeller at 0 to 7000 rpm in a laboratory mock loop. The novelty of this device is the concept of multidirectionality of flow, which is increased without venous obstruction. This is also the first pump that was assessed in a pediatric model (15 kg)^24^; in this in vitro model, the viscous impeller pump-generated flows up to 4.1 L/min and pressure heads of up to 38 mm Hg at 11,000 rpm. It was also associated with low-hemolysis risk. Different from previous devices, Rodefeld et al^25^ used not only computational studies but also in vitro ultrasound measurements to assess the effect of this pump on the hemodynamic performance of the Fontan circulation, showing that a similar pump significantly increases mean velocities of the flow within the total cavo-pulmonary connection. Considering the design of this pump that does not include a cage similar to that of the others, the most recent in vitro analysis on the design by Rodefeld et al^25^ assessed the passive performance of this pump^26^ and found that even if in a failing mode (pump not working), it presents low resistance characteristics with a clinically insignificant pressure drop (<2 mm Hg) in the Fontan circuit, with low-thrombosis risk.

Impella (Abiomed) is a short-term pump already used for left ventricle support. However, the Boston Children’s Hospital group tried to characterize the local response of pressures and flows at the level of the Fontan circuit (specifically, in the left pulmonary artery) to assess the viability of this device (with different calibers) for the cavo-pulmonary application by using a combination of experimental and numeric models of failing Fontan circulation (ie, having high central venous pressures).^27^ According to their in vitro studies, they found that left-sided microaxial pumps, such as Impella, are not well suited for cavo-pulmonary support due to severe flow recirculation (even if lower than in the left sections) and the need for multiple devices in the Fontan circuit. The right-ventricular Impella device provides improved performance by directing flow into the pulmonary artery, resulting in modest decreases in central venous pressure.

**Figure 4:**
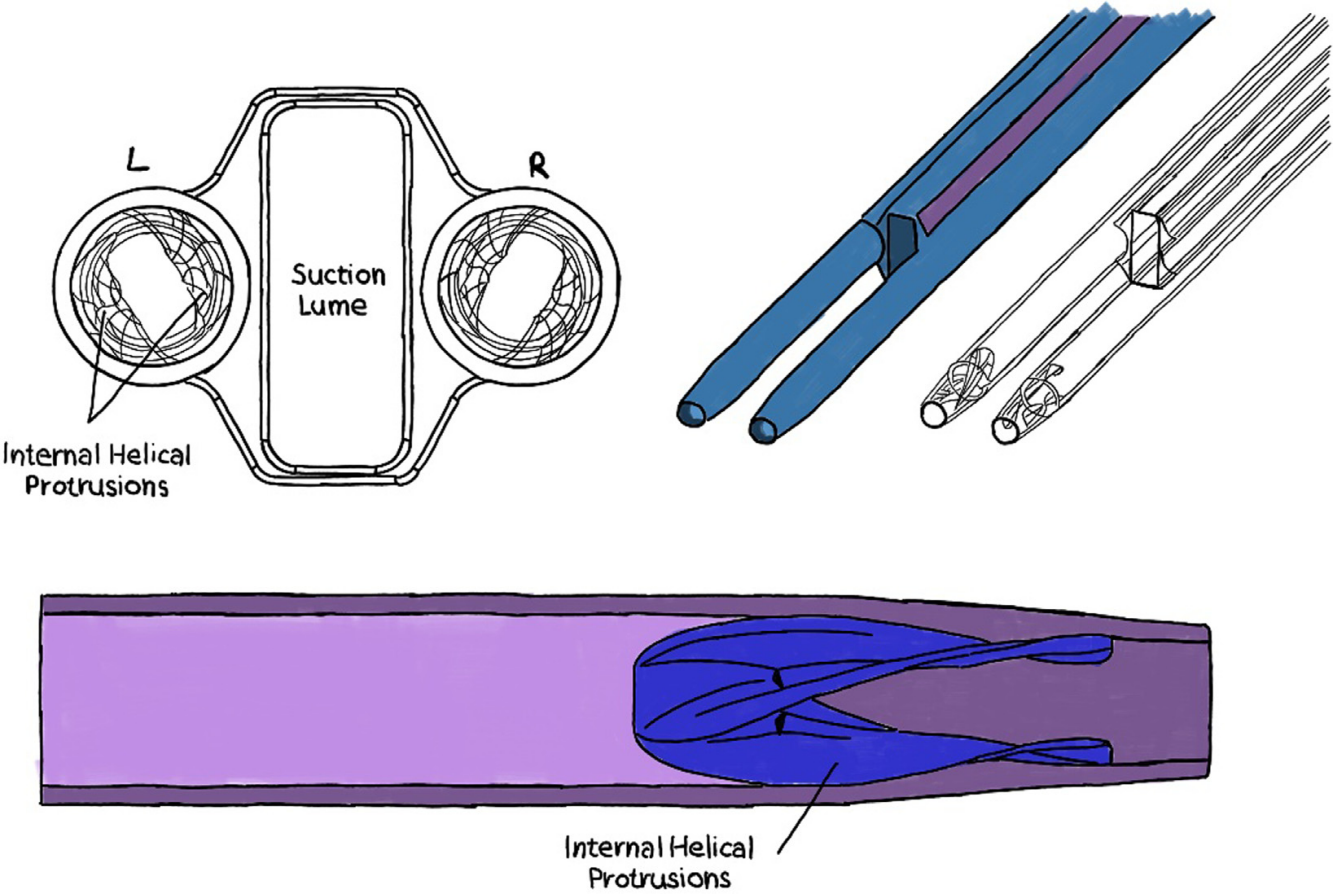
**Multilumen cannula consisting of 2 extruded discharge lumens and 1 suction lumen located in the center**.

In more recent times, a percutaneously inserted multilumen cannula coupled to a commercially available blood pump have been proposed and aimed to provide a pressure gain of 2 to 6 mm Hg.^28^ In this design, the cannula consists of 2 extruded discharge lumens and 1 suction lumen located in the center. The discharge lumens have an internal diameter of 4.5 mm and a tapered inner nozzle diameter of 3.0 mm, whereas the suction lumen has a rectangular cross-section measuring 10.0 mm × 3.75 mm. The exterior cross-sectional dimensions of the cannula are 11.0 × 15.75 mm, which translates to a 33F to 47F size (Figure 4). In the proposed strategy, the cannula is inserted through the right internal jugular vein into the Fontan, where 2 guide wires are then used to guide the discharge lumen nozzles toward their respective pulmonary arteries. Blood from the SVC and IVC is siphoned through the suction lumen into an external centrifugal blood pump before being pumped back to the Fontan through the discharge lumens. Each discharge lumen nozzle contains a set of 2 helical protrusions in the interior to induce a slight swirl to stabilize the flow. The pressurized flow from the pump induces the necessary pressure gradient to overcome downstream pulmonary resistance. This cannula showed promising computational results in pressure gain (4-9 mm Hg) and hemolysis.

## In vivo studies

**Figure 5:**
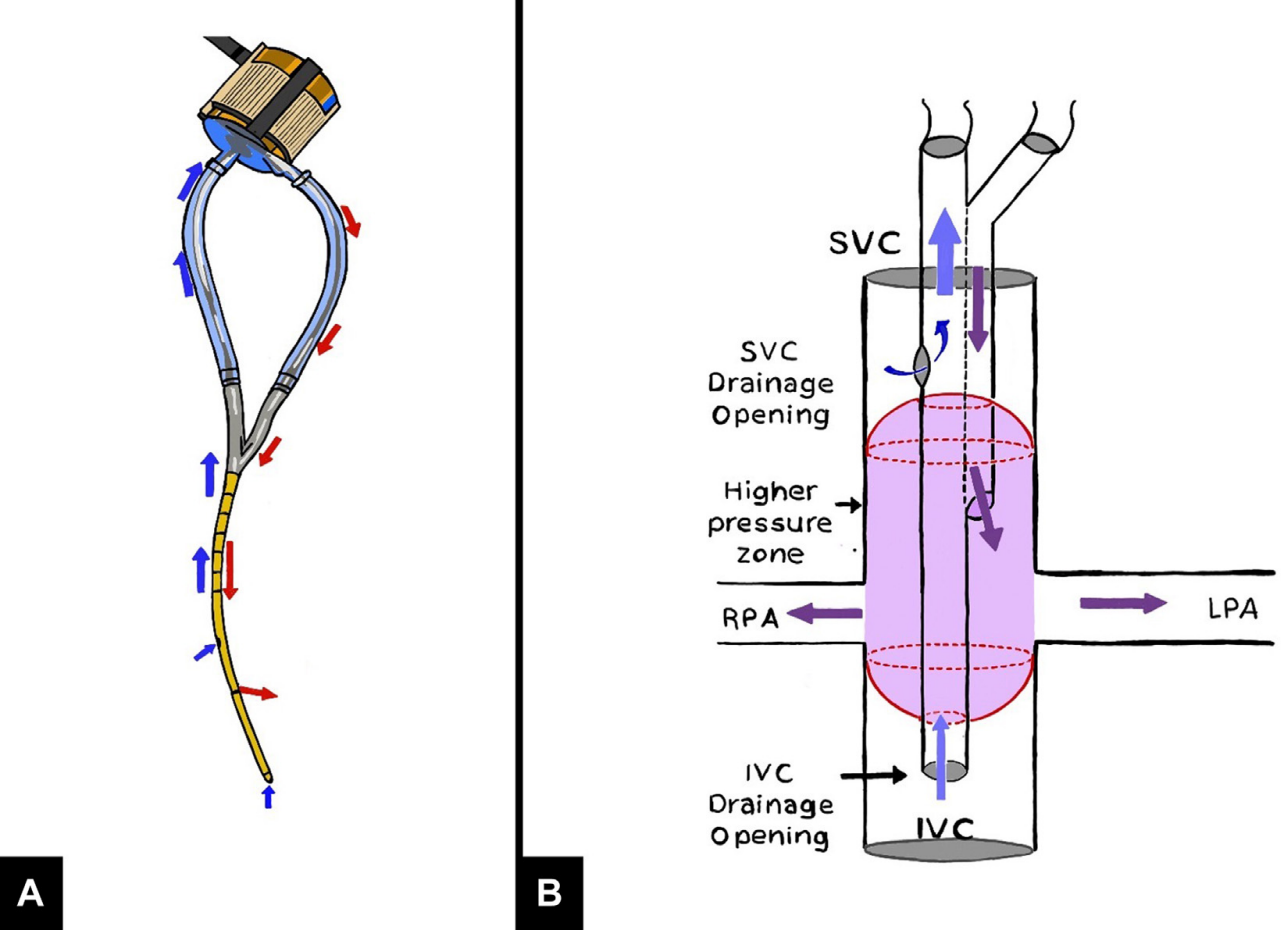
**Percutaneous Wang-Zwische double-lumen cannula (A) and umbrellas added (B).** IVC, inferior vena cava; LPA, left pulmonary artery; RPA, right pulmonary artery; SVC, superior vena cava.

Studies in animal models have been experimented only by a research group led by Dr Dongfang Wang.^29^ The authors created a Fontan sheep model (N = 5) by connecting an extracardiac conduit between the 2 venae cavae, which was then connected to the right pulmonary artery to exclude the right heart.^29^ A percutaneous Wang-Zwische double-lumen cannula (DLC) (commercialized as Avalon Elite; Avalon Laboratories) was coupled with a CentriMag pump (Abbott) to form a cavopulmonary support system (Figure 5A). DLC is a cannula that was first designed and used as right ventricle support in different scenarios.^30^^,^^31^ The DLC was inserted through the right jugular vein and the SVC into the extracardiac conduit with the infusion lumen opening aligned with the right pulmonary artery bridge. Blood was withdrawn from the SVC and IVC through the drainage lumen and pumped into the right pulmonary artery through the infusion lumen, with flow adjusted to 4.0 ± 0.5 L/min. As the percutaneous cannula pumped blood from the vena cava into the pulmonary circulation, functioning as a right heart, the failing Fontan circulation was immediately corrected, with hemodynamics reversed to the level of normal baseline. Because this cannula requires precise outlet alignment to prevent recirculation and can perfuse only one lung, the cannula was modified by adding a pair of membrane umbrellas on both sides of the infusion outlet of DLC.^32^ Two umbrellas were attached, one 4.0 cm above and another 4.0 cm below the infusion opening. Umbrellas were temporarily wrapped and glued to the DLC body to facilitate insertion (Figure 5B). This cannula was successfully tested in 6 adult Fontan model sheep. Alternatively, in more recent times, to avoid this umbrella configuration of the cannula, the same research group hypothesized to use of a bivalved extracardiac conduit as a way to avoid recirculation in the sheep model, maintaining the original DLC cannula without any additional variations.^32^ At 4 L/min pumping flow, the Avalon Elite DLC returned hemodynamics/lactate to baseline levels throughout 6 hours.

## Discussion

Worldwide, over a million children are born each year with congenital heart anomalies. A special heterogeneous subset of this group encompasses babies born with only 1 functional ventricle. Without surgical intervention, this combination of cardiac anomalies is fatal within the first 2 weeks of life. Although the creation of a Fontan circulation allows the majority of patients born with single ventricle congenital heart defects to survive childhood, the cavo-pulmonary total connection represents only palliation, which is associated with increased morbidity and mortality. The reason for this worse outcome is related to the concept of failing Fontan, which is caused by multiple factors. It is well established that patients undergoing this operation have severely decreased systemic venous compliance. This layered physiology results in elevated venous pressures, which are needed to drive pulmonary flow. Even if heart transplantation represents the gold standard for this population, donor shortage significantly limits this source of transplant and imposes the development of viable alternatives.

Current mechanical blood pumps were designed and developed for adult patients with congestive heart failure and to support systemic circulation, not the unique anatomic physiology of the cavo-pulmonary connection. These devices produce pressures exceeding the desired range to be used for cavo-pulmonary support. A pressure augmentation of 2-5 mm Hg may be sufficient to augment the energy required to drive blood flow in the cavo-pulmonary circulation. Veno-arterial extracorporeal membrane oxygenation is used to assist the patient with failing Fontan circulation; however, it is a time-limited strategy, labor intensive, and expensive. A ventricular assistance device (VAD) could also be used, but the Fontan must be taken down and the SVC and IVC must be reconnected for the VAD drainage. Surgical reoperations, especially in the Fontan physiology, are associated with increased surgical complexity and perioperative risks (eg, bleedings and caval and pulmonary damage). To date, the only surgical device used to directly support the cavo-pulmonary circulation is the EXCOR venous cannula (Berlin Heart), which was successfully implanted in a 12-year-old patient as a bridge to transplant.^33^ However, it still requires a surgical reoperation with increased intraoperative and postoperative risks, and it is not a destination therapy. Therefore, the benefit of optimizing venous hypertension and liver dysfunction to perform a successful transplantation is associated with the disadvantage of performing a risky reoperation.

The failing Fontan is primarily related to the absence of a right pump.^5^ However, ∼30% of patients develops systemic ventricle systolic dysfunction^34^^,^^35^; this condition is more frequent in patients with a right-morphology single ventricle, and it is associated to worse outcomes. In fact, systolic dysfunction can increase common atrium filling pressures and impairs postcapillary pressure, finally leading to cavo-pulmonary hemodynamic failure. In this specific scenario, mechanical assist support (MCS) has been widely experienced in Fontan patients with acceptable and promising results over the last 5 years. Data from the ACTION Registry (the first multicenter outcome analysis in Fontan patients undergoing VAD implant)^36^ demonstrated that VAD support can be effective, with ∼80% of patients experiencing a positive outcome at 1 year (70% transplanted and 9% alive on device). Moreover, 70% of patients experienced at least 1 adverse event (primarily neurologic events). All of these neurologic events occurred in patients on an HeartWare Ventricular Assist Device (Medtronic), CentriMag (Abbott), or EXCOR (Berlin Heart). Given the superior hemocompatibility of the HeartMate 3 and with further maturation of the field, the authors expect this will decrease significantly. Many of these patients had improved hemodynamics (decreased Fontan pressures and common atrial pressures) post-VAD and this highlights the concept that VAD therapy might be effective when Fontan failure is due to systolic dysfunction with elevated end-diastolic pressure and low-cardiac output. The most recent analysis of the Society of Thoracic Surgeons Pedimacs and Intermacs Databases on 55 patients who underwent MCS implant showed 6-month overall survival of >70%, similar to patients with biventricular congenital heart disease listed for heart transplantation.^37^ Among these 55 patients, 64% received an implantable VAD (eg, HeartWare, HeartMate 2, and HeartMate 3), 33% a paracorporeal VAD (eg, Berlin Heart), and 1 patient a total artificial heart (eg, CardioWest). More than 80% of these MCS were bridge to transplantation, whereas only 1 patient was a destination therapy. Percutaneous support of Fontan patients has been also reported using an Impella device in the failing systemic ventricle of 10 patients as a bridge to recovery or to decision.^38^ Unfortunately, systemic MCS (both intracorporeal or percutaneous) are associated with an increased risk of thromboembolic ischemic events (eg, brain and GI) and GI bleeding, which might be prevented with a pump limited to the Fontan circulation. Hypothetically, in the presence of systemic ventricle dysfunction, a bi-VAD strategy, including both a percutaneous pump in the systemic ventricle and in the Fontan circulation might restore a more physiologic flow as a bridge to transplantation. Consequently, the development of microinvasive devices, such as percutaneous pumps, is mandatory to limit the surgical risks and systemic cardioembolic events.

The implementation of an ideal blood pump in the cavo-pulmonary circulation should reduce systemic venous pressure, improve transpulmonary blood flow, and improve ventricular filling. Therefore, there are several advantages in creating an artificial intravascular subpulmonary pump: reducing liver congestion (with a consequent better hepatic function, reduced risk of coagulative disease, and hepatorenal syndrome); reducing splanchnic congestion (with lower bowel malabsorption and GI bleeding); reducing venovenous collateral formation (with lower risks of bleeding at the time of heart transplantation); optimizing pulmonary flow and pulmonary vasculature function and consequent improved systemic ventricular filling with better systemic hemodynamics; and improving blood oxygen saturation with better systemic perfusion and reduced risk of systemic-to-pulmonary collaterals formation. In the design of blood pumps, the hydraulic performance is as important as the fluid dynamics and propensity for hemolysis and thrombosis. Blood bag experimentations should be also performed to measure the levels of hemolysis.

Regarding the field of mechanical circulatory support for Fontan physiology, there are presently no medical devices available to support patients who have undergone the Fontan procedure whereas they await donor hearts or to rehabilitate a failing Fontan circulation to optimize chances of heart transplant survival. Current pumps under development include both intravascular pumps and intravascular cannulas with extracorporeal pumps. Intravascular devices have been tested only in vivo in virtual or patient-based models and have shown promising hemodynamic results (Table 1). Differently from left ventricle intravascular pumps (eg, Impella), a cavo-pulmonary pump must take into account different issues: the complex anatomy of the Fontan; the low pressures of the circuit and the risk of vascular collapse; the greater risk of intravascular thrombosis; the complex interaction between SVC and IVC, and between caval and pulmonary district.

**Table 1:** Summary of the main intravascular designs for mechanical Fontan circulation assistance.

Design		Flow rate (L/min)	Pressure gain in the Fontan circuit (mm Hg)	Rotational speed (rpm)	Preclinical evaluation
Collapsible axial flow pump	3-blade impeller	0.5-10	4-22	4000-7000	In vitro
	4-blade impeller	0.5-10	4-28	4000-7000	
Rigid axial flow pump	Impeller with 400° of blade twist	1-5	3-31	6000-8000	In vitro
Viscous impeller pump		0-5	5-20	0-7000	In vitro
		0-4.1	0-38	0-11,000	In vitro
Impella RP (Abiomed)		0.2-1.5	15-42	17,000-33,000	In vitro
Multilumen cannula coupled to a commercially available extracorporeal blood pump		1-4	4-9		In vitro
Wang-Zwische double-lumen cannula (Avalon Elite; Avalon Laboratories) coupled with a CentriMag pump (Abbott)		4.0 ± 0.5			In vivo Fontan sheep model (N = 5)
	Modified cannula with a pair of membrane umbrellas on both sides of infusion outlet of the double-lumen cannula	0-4			In vivo Fontan sheep model (N = 6)

The main limitations of the current devices under investigation are represented by the difficulty of testing these pumps in a Fontan model. To date, the sheep model is the only one that was tested, but the early promising results might not reflect the real failing Fontan scenario, which might be severely influenced by both pulmonary circulation and liver dysfunction. Besides, human applications of these pumps are still to be approved and are essential to establish their safety and efficacy in adapting to this abnormal circulation. While developing these devices, we also remember the potential clinical applications and limits of similar percutaneous pumps: these pumps would be realized for temporary support of the failing Fontan as a bridge to transplantation or total artificial heart implantation. Consequently, patients would be hospitalized, and given the potential for thrombosis (requiring aggressive anticoagulation), hemolysis, or infection (invasive transvenous access), these devices might be used as short-term pumps for a very limited time. In addition, similar devices would remain in place for days, limiting patients’ mobility (increased risk of pump dislodging). Venous system is at lower pressure, consequently any percutaneous device in its position would show a significantly greater risk of thrombosis.

The future of microinvasive intravascular pump design for Fontan support should acknowledge all previous clinical physiologic goals. In addition to an adequate hydraulic performance and to reduce the increased thrombogenicity of artificial materials, regenerative medicine should be used to cover these developing intravascular devices with biocompatible membranes,^39^ to reduce the need for anticoagulation and its consequent risk for the patients. Indeed, their dimensions should not interfere with the potential of additional transcatheter diagnostic or therapeutic procedures on pulmonary arteries.

In conclusion, the current burden of failing Fontan has led researchers to study and develop alternative transcatheter solutions to temporarily support the failing cavopulmonary circulation as a bridge to transplantation in selected cases (Central Illustration). Available devices under development have shown encouraging results regarding both increased flow generated into the Fontan and risk of hemolysis but might start to be evaluated in the clinical setting.

**Central Illustration:**
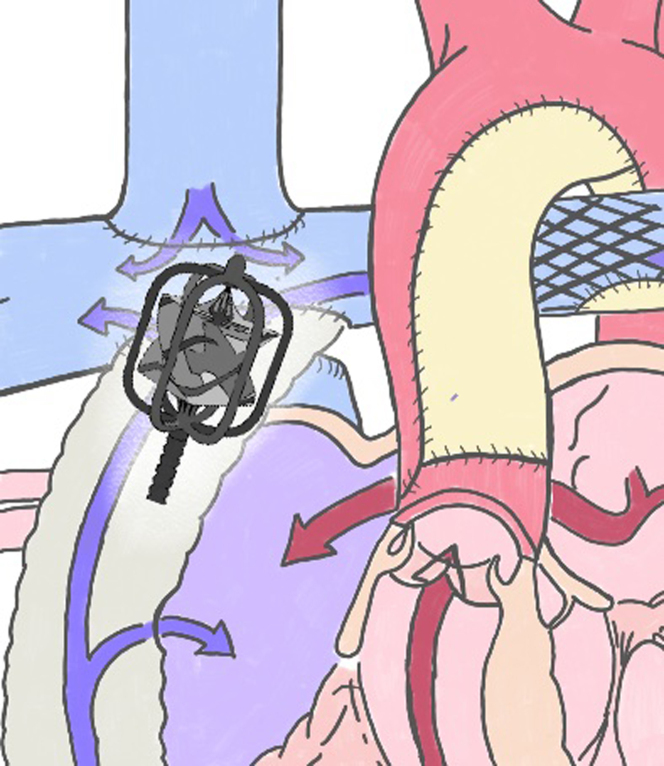
Cavo-pulmonary total connection (Fontan) showing an ideal preclinical prototype of pump inside the circuit.
